# A human antibody specific for SIRPα reprograms macrophages and promotes antibody mediated anti-cancer activity

**DOI:** 10.1371/journal.pone.0321169

**Published:** 2025-05-23

**Authors:** Keifer G. Kurtz, Irina Lebedeva, Stephanie A. Pierre, David Andrew, Yu-Rou Liao, Daisy Ambriz, Olivia Vergnolle, Leyi Shen, Elizabeth Nyakatura, Manuel Baca, David A. Scheinberg

**Affiliations:** 1 Molecular Pharmacology Program, Sloan Kettering Institute, New York, United States of America; 2 Pharmacology Program, Weill Cornell Medicine, New York, United States of America; 3 Tri-Institutional Therapeutics Discovery Institute, Weill Cornell Medicine, New York, United States of America; 4 Tri-Institutional MD-PhD Program, Weill Cornell Medicine, New York, United States of America; 5 Department of Medicine, Memorial Hospital, New York, United States of America; Universite Paris-Saclay, FRANCE

## Abstract

Several T cell immune checkpoint blockade therapies have shown initial successes in multiple cancers. However, significant issues remain, including tumor relapse, severe toxicities, and a lack of efficacy in most patients. SIRPα, commonly known as the “do not eat me signal”, is a monocyte checkpoint cell surface protein. Agents that block the interaction of CD47 with SIRPα have recently shown clinical success in combination with monoclonal antibody therapy to potentiate macrophage phagocytosis of tumors. However, significant toxicities and logistical issues are associated with CD47-targeted agents due to the expression of CD47 on all human cells. In contrast, SIRPα has expression limited to myelomonocytic cells, meaning highly specific SIRPα blocking agents might reduce these toxicities and avoid the target antigen sink. Herein, we generated a high affinity and highly specific SIRPα-targeting monoclonal antibody, F05, that has enhanced SIRPα binding and reduced SIRPβ and SIRPγ binding capacity when compared to other available SIRPα antibodies. Furthermore, we show F05 reprograms immunosuppressive macrophages toward a phagocytic profile *in vitro*. F05 demonstrated efficacy in solid tumor animal models, providing a rationale for further development of the antibody.

## Introduction

Multiple immune checkpoint blockade inhibitors have been approved since 2011 as anti-cancer agents [1]. These approved agents all target proteins associated with improving T-cell function, such as CTLA-4, PD1 and PD-L1, Tim 3, and Lag3 [1]. Only a subset of patients respond well to these therapies [2]. This lack of uniform success has also prompted exploration of other immune checkpoints affecting tumor-associated immune cells that are not T cells.

The CD47-SIRPα axis has recently emerged as a key pathway for development of a new class of immune checkpoint cancer-targeting agents. CD47 is ubiquitously expressed, but many tumor cells overexpress this protein as a protection mechanism. In contrast, SIRPα has expression limited to myelomoncytic cells, most prominently on macrophages. This axis is known as the “do not eat me” pathway because engagement of CD47 with SIRPα results in an inhibitory signal for macrophage phagocytosis through activation of the ITIM domains of SIRPα, resulting in downstream inhibition of actin-myosin engagement and therefore reduced macrophage phagocytosis [3–6].

In pre-clinical studies, blockade of the CD47-SIRPα alone results in minimal anti-tumor effects [7]. However, this strategy in combination with a tumor-opsonizing monoclonal antibody provides the macrophages a signal to phagocytose, and has proven to be highly effective. Numerous studies have demonstrated the anti-tumor benefit with this combination strategy *in vivo*, including a triple combination therapy approach with CAR T-cells [8]. However, the vast majority of these agents bind CD47, which has resulted in severe toxicities in the clinic due to reduction of red blood cells and platelets, which also express high levels of CD47 [9]. In addition, the widespread expression of CD47 requires large and frequent doses of the inhibitors to saturate the CD47 antigen sink.

The clinical limitations of CD47-targeting agents has prompted a recent shift towards development of SIRPα targeting agents with a goal of reducing systemic toxicities and the antigenic sink. In the pre-clinical setting, several SIRPα targeting monoclonal antibodies have been efficacious in vivo in several cancer types. Additionally, a high-affinity peptide mimetic of the CD47 extracellular domain has been developed, but no in vivo efficacy has been described [10]. Five SIRPα-targeting antibodies have been described in the preclinical setting: 1H9, KWAR23, BR105, AL008 and BYON4228 [11–15]. In the clinic, only 1H9 has advanced as GS-0189. While well-tolerated in a phase I trial in combination with Rituximab for treatment of patients with relapsed or refractory non-Hodgkin’s lymphoma, GS-0189 was discontinued for further clinical development.

While targeting SIRPα has shown success in eliminating the thrombocytopenia associated with CD47-targeting agents, the strategy still presents a risk through potential binding of these antibodies to SIRPβ and SIRPγ, which have high sequence homology to SIRPα in the extracellular domain [16]. The function of both SIRPβ and SIRPγ are not well described; however, initial characterization of SIRPγ has revealed a role in promoting the chronic activation and migration of T-cells and promoting stem-like characteristics of tumor cells [17–20]. Therefore, potential blockade of SIRPγ may result in the inhibition of tumor-associated T cells, which would reduce the therapeutic effect of SIRPα targeting antibodies. In view of these deficiencies, there is still a demonstrated need for effective, high affinity *SIRPα specific* agents.

We sought to develop a SIRPα specific antibody that enhances the antitumor effect of mAb therapy in both hematopoietic cancers and solid tumors. F05 was identified as a high affinity SIRPα antibody clone with binding capacity for both SIRPα V1/V1 and V2/V2. Importantly, F05 does not bind SIRPγ on human T cells, which mitigates the risk of potential inhibition of tumor infiltrating lymphocytes (TIL) or other activated T cells systemically. We demonstrated that F05 reprograms human macrophages *in vitro* towards a phagocytic phenotype, which resulted in enhanced anti-tumor effects in two models. Importantly, in three models involving both hematopoietic cancers and solid tumors, we show that F05 in combination with mAb has additive efficacy *in vivo* when compared to mAb treatment alone. We propose that F05 is a promising SIRPα specific agent with the potential for treatment in a variety of cancer types.

## Materials and methods

### Cell lines

Ba/F3, a murine interleukin-3 dependent pro-B cell line (DSMZ cat #ACC 300) and its derivative overexpression huSIRPα was maintained in 95% RPMI 1640 supplemented with 5% h.i. FBS and 10 ng/mL mouse IL-3. T-ALL Jurkat cells and human monocytic cells U937 and THP-1 were obtained from ATCC and cultured as recommended by vendor. Raji and Skov3 tumor cells expressing GFP/FireFly Luciferase were generously provided by the lab of Renier Brentjens. All cell lines used in this study were maintained at 37°C in a humidified atmosphere containing 5% CO_2_ and monitored periodically to ensure the absence of mycoplasma.

### Generation of huSIRPα specific human mAbs

huSIRPα-binding mAbs with human variable regions were developed by standard hybridoma technology using AlivaMab Mouse (Ablexis LLC) transgenic mouse strains that produce chimeric human-mouse antibodies consisting of human Fab domains and a murine Fc domain. Two cohorts of AlivaMab mice were immunized accordingly with recombinant full length huSIRPα protein fused to a poly-histidine tag, or Ba/F3 cells stably expressing huSIRPα. Both cohorts of mice were primed using His-tagged full length recombinant huSIRPα protein, and then mice were boosted at 3-week intervals for at least 3 times, protein (1^st^ cohort) or OE cells IP (2^nd^ cohort). Mouse sera was tested via enzyme-linked immunosorbent assay (ELISA) against huSIRPα-His and irrelevant His-tagged protein as well as via flow cytometry using Ba/F3 cells (overexpressing huSIRPα and WT) and mice exhibiting the highest titres in both assays were acute boosted prior to electrofusion of splenocytes with SP2/0 cells for hybridoma generation. Hybridoma supernatants were screened for reactivity via ELISA (huSIRPα-His protein) or Mirrorball (microplate cytometry) assay using OE cells. huSIRPα-specific clones were screened additionally for CD47 blocking ability via flow cytometry. For sequencing of successful clones, RNA was isolated from hybridoma cells, and immunoglobin cDNA was synthesized using a deoxythymidine oligonucleotide primer and reverse transcriptase. Variable regions were PCR-amplified using AlivaMab™-specific set of primers, and then sequenced via standard Sanger method.

### Recombinant production of SIRPα-specific human mAb

For recombinant production of F05 and 1H9 (the reference antibody), the antibodies were formatted as mouse IgG2a/kappa, possessing the L234A, L235A, P329G mutations to disrupt any antibody effector function. The production of all antibodies was carried out at GenScript™.

Briefly, antibody sequences were generated by gene synthesis and cloned into cytomegalovirus promoter-driven expression vectors. All proteins were expressed by transient co-transfection in CHO-Express cells and purified by MabSelect™ PrismA affinity chromatography. The purified protein was analyzed by SDS-PAGE, SEC-HPLC, and intact mass to determine the purity and molecular weight.

### Cell-based binding of huSIRPα Abs

To assess the binding of Abs to cell-expressed huSIRPα, Ba/F3 cell line overexpressing huSIRPα, U937 or THP-1 cell lines were used. For binding to cell-expressed SIRPγ, Jurkat T-ALL cell line or human PBMCs were used. Cells were blocked with Fc fragment of hu IgG1 (Jackson Immuno Research Laboratories) for 20 min at room temperature and incubated for 1 h on ice with serial dilutions of huSIRPα-antibodies in binding buffer containing 1.5% BSA (w/v) in PBS without Ca^2+^ and Mg^2+^ (8 points, 1:5 dilutions, starting concentration 20µg/mL). The cells were then stained with Alexa Fluor® 647 AffiniPure goat anti-mouse IgG, Fcγ fragment specific secondary antibody (Jackson Immuno Research Laboratories). The cells were analyzed via flow cytometry (GUAVA flow cytometer, Luminex Corp.) using FlowJo Software (v10.8.1 for Mac OS X; Becton Dickinson). The binding was assessed as the geometric mean fluorescence intensities (GMFI) and normalized to GMFI of cells stained with secondary antibody alone. EC_50_ binding values were calculated using a nonlinear four parameter variable slope fit model (Prism v8; GraphPad).

### Binding to human, mouse and cynomolgus monkey SIRPα, huSIRPβ and SIRPγ via ELISA

Human SIRPα (ACRO Biosystems), mouse or cynomolgus monkey SIRPα, huSIRPβ, huSIRPg (all from R&D Systems) were adsorbed to high-binding microtiter plates (Corning) at 2 µg/mL in PBS, blocked with SuperBlock™ (PBS) blocking buffer (ThermoFisher Scientific) and incubated with serially diluted indicated mAbs (7 points of 1:4 dilutions, starting concentration 10µg/mL). The samples were then incubated with HRP-labeled AffiniPure Goat anti-mouse IgG, Fcγ specific secondary Ab (Jackson Immuno Research). TMB Substrate Solution (ThermoFisher Scientific) color development was stopped by adding 1 M sulfuric acid and absorbance was read at 450 nm on a microplate reader (Molecular Devices). A dose response curve was fitted by 4‐parameter logistic (4PL) regression (Prism V.8; GraphPad).

### Antibody affinity measurement

Affinity experiments were performed on an Octet RED96 (ForteBio) at 25°C. The test Abs were captured onto anti-mouse IgG Fc capture (AMC) biosensors (ForteBio). Measurements were made with serial dilutions of human huSIRPα -His fusion proteins (ACRO Biosystems). The association of the antigen was measured for 40 s, followed by a dissociation step for 100 s. Curve fitting was performed using a 1:1 binding model and the ForteBio data analysis software V.9.0 (ForteBio).

### Blocking human CD47 binding on huSIRPα - expressing cells

Ba/F3 cells overexpressing huSIRPα huSIRP were incubated for 1 hr on ice with serial dilutions of tested antibodies (1:5 dilutions, eight points, starting concentration 50µg/mL) in the absence or presence of human IgG1 Fc-tagged human CD47 (R&D Systems). Binding of CD47 on the cells was measured by adding Alexa Fluor® 647 AffiniPure goat anti-human IgG, Fcγ fragment specific secondary antibody (Jackson Immuno Research) and analyzed by flow cytometry. Binding was normalized to the mean fluorescence intensity of CD47- huSIRPα binding in the absence of SIRPα-specific antibodies.

### Blocking human CD47-induced SIRPα signaling

Jurkat cells (Jurkat-SIRPα) expressing a split β-galactosidase enzyme, wherein the enzyme acceptor is tethered to SHP1 and the enzyme donor is tethered to the ITIM’s of SIRPα, was purchased from Eurofins (#93-1135Y19-00130). Raji tumors were utilized as CD47-presenting cells. Jurkat-SIRPα and Raji cells were co-cultured at a 1:1 ratio in the absence or presence in a range of mAb doses. Luminescent signal was normalized to the untreated condition.

### T cell function and proliferation

Human PBMC’s were collected from healthy donors given informed consent on MSK IRB approved protocols and treated with IL-2 (200 IU’s/mL) with or without 10 nM of 1H9 or F05 antibodies on day 0. On day 3, PBMC’s were collected, and T-cells were identified based on CD45^+^, CD3^+^ expression using flow cytometry. Dead cells were removed using Viakrome. CD25-BUV395, CD69-PE eFluor 610, and Ki67-FITC geometric means were used to determine MFI differences between the untreated condition and the 1H9 treated and F05 treated samples. Additionally, PBMC’s were collected and treated with IL-2 (200 IU’s/mL) and ImmunoCult™ Human CD3/CD28 T Cell Activator (25ul/ml) with or without 10 nM of 1H9 or F05 antibodies on day 0. On day 3, PBMC’s were collected, and T-cells were identified based on CD45^+^, CD2^+^ expression using flow cytometry. Dead cells were removed using Viakrome. CD25-BUV395, CD69-PE eFluor 610, and Ki67-FITC geometric means were used to determine MFI differences between the untreated condition and the 1H9 treated and F05 treated samples.

### SIRPα and CD47 expression on tumor cells

50,000 Raji-GFP FireFly Luciferase or 50,000 MUC 16 + SKOV3-mcherry Gaussia Luciferase cells were collected and measured for SIRPα-expression using a two-step stain. Anti-human SIRPαClone 15–414 was used as the primary antibody, followed by anti-mouse IgG PE antibody as the detection antibody. A mouse IgG2A antibody was used as the isotype control. For CD47 expression, 50,000 cells (Jurkat, Raji-GFP FireFly Luciferase, MUC 16 + SKOV3-mcherry Gaussia Luciferase, or THP1 cells) were collected and measured using a one-step stain by flow cytometry. A mouse anti-human CD47-APC (clone: CC2C6) antibody was used for detection of surface-bound CD47 along with an isotype control (mouse IgG-APC).

### In vitro M2 Macrophage Phagocytosis and Reprograming

CD14 + PBMC’s were isolated from healthy donors given informed consent on MSK IRB approved protocols using MACS based bead separation (Miltenyi Biotech, #130-050-201). Human cells were obtained over a period from March 1, 2022 until June 30, 2023. CD14 + PBMC’s were stimulated with 20 ng/ml M-CSF (Sigma-Aldrich, #M6518) for 4 days, followed by additional cytokine supplementation for 4 days with 20 ng/ml IL-4, IL-6, and IL-13 (R&D Systems, #204-IL, #7270-IL, #213-ILB). Cytokines were replenished every two days. Following huM2 generation, macrophages were dissociated using Corning CellStripper (#25–056-CI) and co-cultured with either Raji or Skov3 tumor cells transduced to express GFP/FireFly Luciferase. mAb’s were also added at the indicated concentrations. Tumor cell lysis was determined by normalization to the untreated condition. mAb-induced macrophage reprogramming was measured via flow cytometry using a Miltenyi MACSQuant16. Tumor cells were identified by GFP expression. M2’s were identified using an antibody targeted to MHC II and lack of GFP expression. Antibodies targeted to CD80, CD86, CD163, and CD206 were used to determine the relative expression of these markers and normalized to the untreated condition.

### In vivo experiments

All animal experiments were performed under a protocol approved by Memorial Sloan Kettering Cancer Center’s Institutional Animal Care and Use Committee (protocol 96-11-044). To evaluate the efficacy of F05 in combination with Rituximab, NSG mice (n = 5 per group) were engrafted s.c with a mixture of huM2’s (1x10^5^, one donor) and Raji tumor cells (2x10^6^) engineered to express GFP/FireFly Luciferase. To evaluate the efficacy of F05 in combination with Traztuzumab, NSG mice (n = 4 per group, PBS group n = 2) were engrafted s.c with a mixture of hu M2’s (1.5x10^5^, one donor) and Skov3 tumor cells (3x10^6^) engineered to express GFP/FireFly Luciferase. Monoclonal antibody therapy was administered via i.p injections at the doses and time points indicated in each experiment. Tumor burden was measured by calipers. Survival analysis was performed using Kaplan-Meyer statistical analysis. Surrogate markers for death were used, and not death, as an endpoint. These included weight loss of >20%, tumor size greater than 2 cm^2^, tumor necrosis or bleeding, or severe morbidity (ruffled fur, decreased mobility, feeding or grooming, hunched posture, paralysis, bleeding, severe diarrhea) restricting normal activities of the mice as assessed daily by the research staff and veterinarians. Mice with large tumors often had other signs of morbidity that required euthanasia. Mice are examined 7 days a week for the appearance of distress or injury. Animals meeting the predetermined criteria above are euthanized using CO_2_ inhalation. Euthanasia was performed within 24 hours of notification of the surrogate marker, or sooner if warranted. A fraction of animals died before euthanasia could be performed. Anesthetic drugs are used to sedate or put mice to sleep before procedures involving bleeding. Antibiotics are given for rashes. Experiments were terminated when the last living mouse was sacrificed (for morbidity or tumor size) or at 100 days if not reached. All investigators are required to pass animal use and care training courses administered by the MSK IACUC before handling mice.

## Results

### *Binding Specificity to SIRP* protein

**Fig 1:**
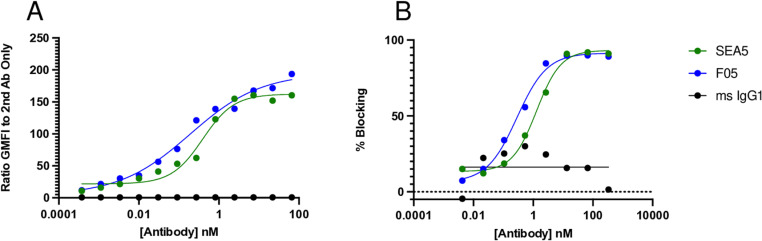
F05 demonstrates binding to cell-expressed huSIRP⍺ and blockade of CD47 binding to huSIRP⍺. A.) Ba/F3 cells overexpressing huSIRP⍺ were incubated with various concentrations of mAb’s followed by anti-mouse IgG Alexa647 for detection by flow cytometry. B.) Ba/F3 cells from (A) were incubated with various concentrations of mAb’s to block binding of soluble CD47-Fc. Percent blocking was detected using an anti-human IgG Alexa647 by flow cytometry.

Ba/F3 cells transduced with full-length huSIRPα tagged with a poly-histidine tag were used to immunize mice for generation of mAb. Chimeric human-mouse antibodies with human Fab and murine Fc domains were generated using hybridoma technology. Recombinant clones were generated with mutated Fc regions to prevent antibody effector function. One clone, F05, was identified to have similar binding avidity as a positive control commercial anti-SIRPα antibody, SEA5, to Ba/F3 cells overexpressing huSIRPα (F05 EC50 = 0.19 nM, SEA5 EC50 = 0.39 nM, Fig 1A). Additionally, F05 blocked the binding of soluble huC47 similarly to SEA5 (F05 IC50 = 0.29 nM, SEA5 IC50 = 1.30 nM, Fig 1B).

Solid-phase ELISA with huSIRPα was used to compare the binding of F05 with 1H9, a commercial anti-SIRPα antibody used in a clinical trial. F05 bound huSIRPα with low-nanomolar affinity (EC50 = 1.03 nM), but lower avidity than 1H9 (EC50 = 0.20 nM). (S1 Fig A in S1 File). F05 did not bind mouse SIRPα but showed similar levels of binding as 1H9 to cynomolgus-derived SIRPα (S2 Fig A and B in S1 File). Next, the cross reactivity of F05 to SIRPγ was tested using solid-phase ELISA; F05 showed ~ 5x decreased binding to huSIRPγ compared with 1H9 (F05 EC50 = .11 nM, EC50 1H9 = .03 nM, S1 Fig B in S1 File). Furthermore, F05 also showed a similar decrease in binding as compared to 1H9 in to huSIRPβ (F05 EC50 = .019 nM, 1H9 EC50 = 0.02 nM, S2 Fig C in S1 File). Taken together, these data indicated F05 is a potent binder to huSIRPα with reduced binding to other SIRP proteins, resulting in an advantage for potential minimization of off-target effects.

**Fig 2:**
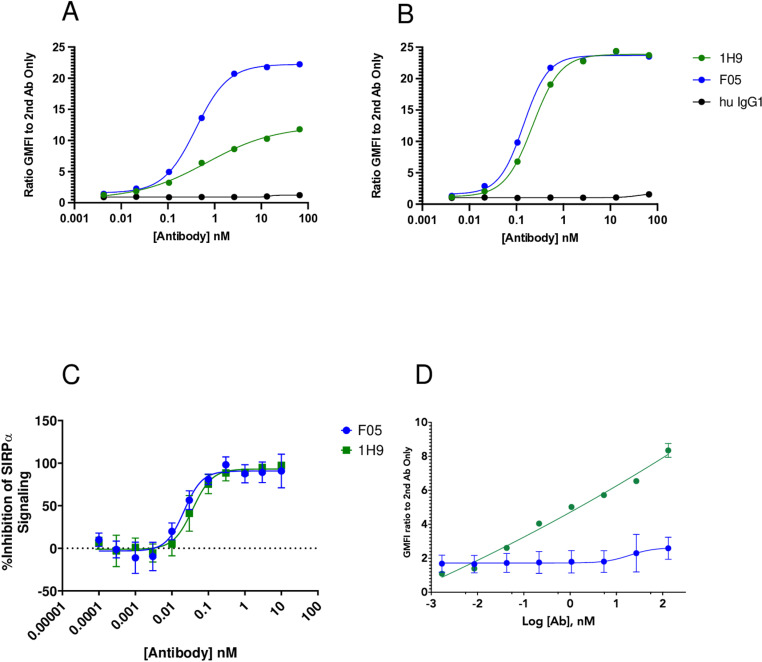
F05 demonstrates pan-SIRP⍺ allele binding, induces blockade of SIRP⍺ signaling, and minimally engages T-cell SIRPʏ. A.) THP1 cells expressing SIRP⍺ V1/V1 variants were incubated with various concentrations of mAb’s. Binding was detected using an anti-mouse IgG Alexa647 by flow cytometry B.) U937cells expressing SIRP⍺ V2/V2 variants were incubated with various concentrations of mAb’s. Binding was detected using an anti-mouse IgG Alexa647 by flow cytometry C.) Jurkat-SIRP⍺ reporter cells were co-cultured with CD47-presenting Raji tumor cells with various concentrations of mAb’s to determine the level of downstream signaling inhibition. D.) Purified human T cells were incubated with either 1H9 or F05 antibodies. Detection of mAb binding was determined using goat anti-human IgG Alexa647 by flow cytometry.

To further evaluate F05, we tested the ability of F05 to bind human cells expressing the two different SIRPα alleles. F05 showed similar levels of binding to THP1 cells (expressing SIRPα V1/V1 variant) as 1H9 (F05 EC50 = 0.40 nM, 1H9 EC50 = 0.68 nM, Fig 2A). This result was similar in U937 cells (expressing SIRPα V2/V2 variant) (F05 EC50 = 0.14 nM, 1H9 EC50 = 0.22 nM, Fig 2B).

### Blockade of signaling

Next, the potency of F05 to block CD47-induced signaling through SIRPα in cells was tested using a combination of CD47-presenting Raji tumor cells and Jurkat cells transduced to express a SIRPα reporter (Eurofins PathHunter Jurkat SIRPα Signaling Bioassay Kit). F05 demonstrated dose-dependent blockade of CD47-induced SIRPα signaling that was comparable to 1H9 (Fig 2C). We also tested the ability of 1H9 and F05 to alter T-cell function and proliferation in PBMCs either unstimulated or after CD3/CD28 stimulation. CD25 and CD69 (activation) and Ki67 (proliferation) levels changed minimally in CD3^+^, CD45^+^ PBMC’s after treatment with the antibodies, consistent with previous work with antibodies to SIRPs[18,20] (S3 Fig in S1 File). These data further demonstrated the utility of F05 as a specific SIRPα binder and CD47 functional blocker, without significant inhibitory or activating effects on T cell activation and proliferation.

### Anti-cancer cell activity

**Fig 3:**
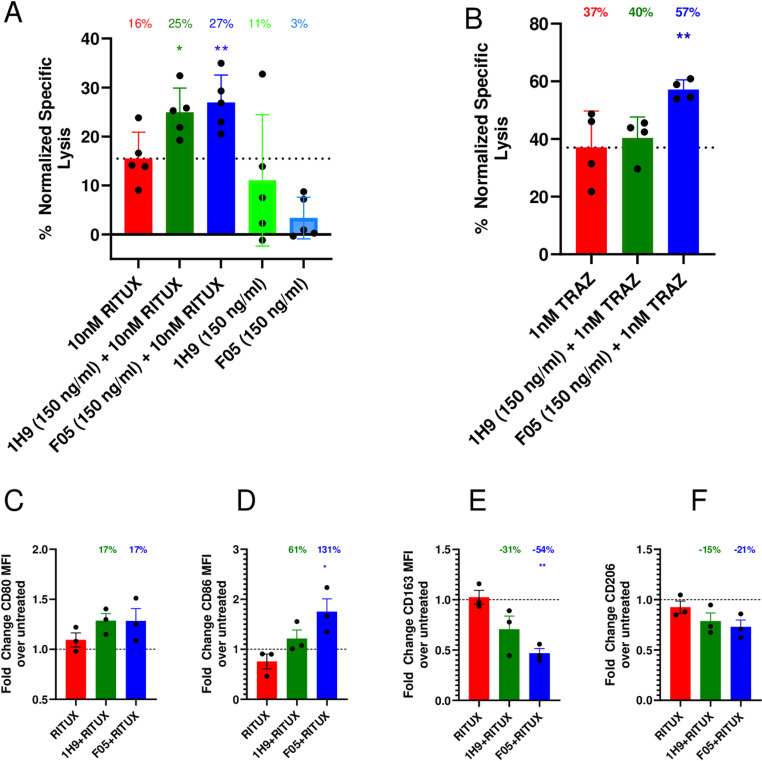
F05 demonstrates additive anti-tumor effects in combination with mAb therapy and reprograms M2 macrophages towards a phagocytotic phenotype. A.) Human M2 macrophages (2.5x10^4^) were co-cultured with Raji tumor cells (5x10^4^) and exposed to mAb’s for 4 hours before Raji luminescence was quantified (two tailed t-test; 1H9, p = 0.021; F05, p = 0.011). Data shown is from 5 donors. B.) Human M2 macrophages (2.5x10^4^) were co-cultured with SKOV3 tumor cells (2.5x10^4^) and exposed to mAb’s for 4 hours before Skov3 luminescence was quantified (two tailed t-test; 1H9, p = 0.66; F05, p = 0.02). Data shown are from 4 donors. C-F.) Quantification of CD80,CD86,CD163 and CD206 MFI by flow cytometry in three donors in experiment as performed in (A). One-way ANOVA demonstrates no significant changes in CD80 or CD206 MFI in either F05 or 1H9 when compared to RITUX treatment. CD86 MFI is significantly increased with F05 treatment (p = 0.0216, 131% increase), but not 1H9 (p = 0.2519, 61% increase). CD163 MFI is significantly decreased with F05 treatment (p = 0.0089, 55% decrease), but not 1H9 (p = 0.0828, 31% decrease) when compared to RITUX.

CD47-SIRPα checkpoint blocking therapies have shown minimal anti-tumor efficacy when used alone as a monotherapy, yet profound activity in combination with therapeutic human mAb. Therefore, we next tested the opsonization effects in vitro of F05 in combination with two widely used tumor-opsonizing clinical antibody therapeutics, Rituximab and Trastuzumab. We generated human M2 macrophages from blood derived CD14 + monocytes and co-cultured them with either CD20 + Raji tumor cells or MUC16 + SKOV3 tumor cells that were both transduced with FireFly Luciferase for measurement of tumor cell viability. As expected, F05 and 1H9 displayed minimal levels of anti-tumor effect when tested as a monotherapy (Fig 3A). However, both F05 and 1H9 in combination with Rituximab demonstrated significantly high levels of macrophage-induced phagocytosis of Raji tumor cells (Fig 3A). F05 also promoted high levels of opsonization of the solid tumor ovarian cancer cells, SKOV3, in combination with Trastuzumab *in vitro*. F05 in combination with Trastuzumab demonstrated significantly higher levels of activity when compared to Trastuzumab as a monotherapy (Fig 3B). Interestingly, 1H9 in combination with Trastuzumab demonstrated minimal enhanced tumor cell destruction. Importantly, MUC16 + SKOV3 or Raji tumor cells express negligible levels of SIRPα and do not function as an antigen sink for these antibodies (S4 Fig in S1 File). We also confirmed CD47 is expressed at high levels on these cell types, thereby confirming blockade of CD47-SIRPα signaling in these experiments (S4 Fig in S1 File).

### Macrophage reprogramming

CD47-SIRPα blockers have previously been shown to not only induce macrophage phagocytosis, but to also reprogram immunosuppressive macrophages from an M2 to M1 state. Hence, we measured the amount of CD80 and CD86, M1 markers, and CD163 and CD206, M2 markers, on macrophages treated with SIRP mAb and Rituximab in three representative samples from the experiments shown in Fig 3A. Only F05, and not 1H9, demonstrated significantly increased levels of CD86 and lower levels of CD163 when compared to Rituximab monotherapy, indicating the dual mAb therapy reprogrammed the macrophages to an M1-like phenotype. (Fig 3D,E, Fig. S5C-D in S1 File). Non-significant changes were observed in CD80 and CD206 treatment with both 1H9 and F05 (Fig 3C, F, Fig. S5 Fig B to E in S1 File). These findings demonstrated that F05 can be utilized to enhance the anti-tumor effects of opsonizing mAb therapy for both lymphomas and solid tumors.

### Mouse models of cancer therapy

**Fig 4:**
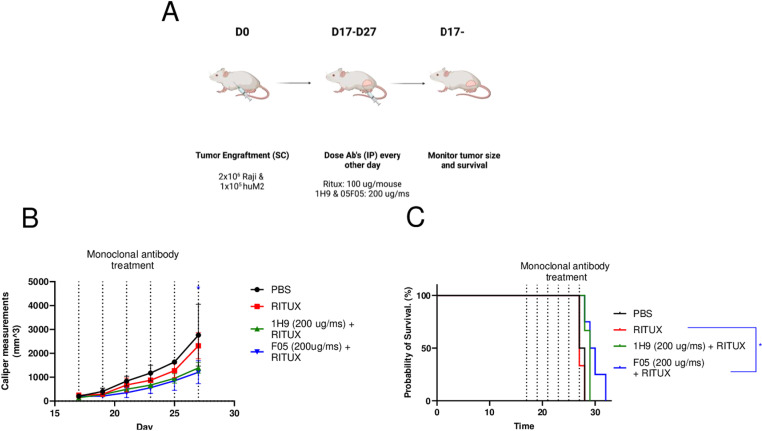
F05 demonstrates additive anti-tumor effects in combination with mAb therapy *in vivo.* A.) Scheme. A mixture of Raji tumor cells (2x10^6^) and human M2 macrophages (1x10^5^) were injected on D0 and mAb therapy was started on D17 post tumor injection every other day for a total of 6 doses. B.) Tumors as measured by calipers overtime (two-tailed t-test; F05, p = 0.032; 1H9, p = 0.052) C.) Survival curve analysis of (A-B). Control group mice with PBS or Rituximab were sacrificed due to tumor size and treatment group mice were sacrificed for surrogate death endpoints. (Mantel Cox log-rank: F05 vs RITUX, p = 0.03; 1H9, p = 0.063). Mean + /- SD are plotted for all.

We next tested the *in vivo* efficacy of F05 in combination with Rituximab in vivo. Because neither F05 and 1H9 bind the murine SIRPα, we generated models in which subcutaneous tumors are formed with a combination of Raji tumor cells and human M2 macrophages as effectors. We dosed F05 and 1H9 in combination with Rituximab, or Rituximab alone, every other day starting on Day 17 post-tumor engraftment (Fig 4A). Mice dosed with F05 plus Rituximab demonstrated significantly reduced tumor volume, as measured by caliper, compared to mice treated with Rituximab alone at Day 27 post-tumor engraftment (two-tailed t-test; F05, p = 0.032; 1H9, p = 0.052, Fig 4B). Importantly, the F05 combination treatment also demonstrated significantly increased survival compared to Rituximab monotherapy (Mantel Cox log-rank: F05 vs RITUX, p = 0.03; 1H9, p = 0.063, Fig 4C). We further evaluated the efficacy of F05 combination treatment in a less aggressive tumor model in which antibodies were dosed starting on Day 11 post tumor engraftment (S6 FigA in S1 File). A modest, although not significant decline, in tumor burden was observed in both the F05 and 1H9 combination treatment when compared to Rituximab monotherapy (S6 Fig B in S1 File). Minimum survival benefit was observed in this model with combination treatment (S6 Fig C in S1 File), indicating these antibodies may have enhanced efficacy for treatment of larger, more aggressive tumors.

**Fig 5:**
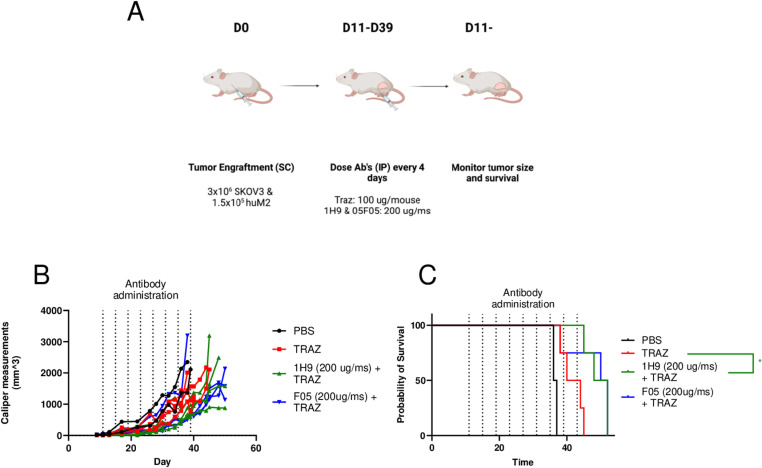
F05 demonstrates additive anti-tumor effects in combination with mAb therapy in a solid tumor model. A.) Scheme. A mixture of SKOV3 tumor cells (3x10^6^) and human M2 macrophages (1.5x10^5^) were injected on D0 and mAb therapy was started on D11 post tumor injection and performed every 4 days for a total of 8 doses. B.) Tumors as measured by calipers overtime. C.) Survival curve analysis of mAb’s compared with Trastuzumab alone. Nine animals were were sacrificed due to tumor size and the rest were sacrificed for surrogate death endpoints. (Mantel Cox log-rank: F05 vs TRAZ; p = 0.078;1H9 vs TRAZ, p = 0.017).

Last, we investigated the potential for F05 in treatment of solid tumors using mice engrafted with a mixture of human M2 macrophages and SKOV3 tumors cells (Fig 5A). While significant tumor burden was only modestly reduced by the treatments (Fig 5B), likely due to the aggressive nature of this model, differences in survival of mice treated with mAb combination therapy were seen (Fig 5C). In conclusion, F05 showed additive effects with therapeutic mAb in three different animal therapy models.

## Discussion

Herein, we demonstrate that a new mAb, F05, selectively binds SIRPα over SIRPβ and SIRPγ, binds both SIRPα variants V1/V2 and importantly, does not bind SIRPγ-expressing human T-cells. Because of the important role of intra-tumoral T cells in eliminating tumor cells, and the potential for reduction of T-cell function by engagement of SIRPγ, the lack of binding of F05 to human T cells provides a distinct advantage over other available agents.

We selected F05 for further characterization in a macrophage phagocytosis assay using human-derived M2 macrophages co-cultured with either Raji lymphoma cells or SKOV3 ovarian cancer cells. In combination with either Rituximab or Trastuzumab, we show that F05 has additive anti-tumor effects with mAb therapy when compared to mAb therapy or F05 alone. Indeed, F05 shows little anti-tumor effect alone, as widely demonstrated across all CD47-SIRPα targeting agents. Because both Rituximab and Trastuzumab are clinically approved agents, we suggest that these findings may provide a superior rationale for translation of F05 into the clinic with these agents compared to other combination therapies due to its minimal binding to other SIRP proteins.

To further demonstrate the potential clinical application of F05 in combination therapy, in three models, F05 has enhanced efficacy when compared to mAb treatment alone. Because F05 does not bind mouse-derived SIRPα, we generated tumors in vivo with a combination of human M2 macrophages mixed with either Raji or SKOV3 human tumor cells. In a large tumor burden model using Raji subcutaneous engraftment, F05 significantly reduced tumor burden and enhanced mouse survival, albeit very briefly compared to the Rituximab monotherapy. In a mouse model bearing smaller tumors, F05 reduced tumor burden and enhanced mouse survival, but non-significantly. While these models do not represent a more disseminated lymphoma, the solid tumor models are arguably harder to treat and strongly indicate the utility of F05 as an agent for treatment of lymphoma. Furthermore, we tested the efficacy of F05 for treatment of SKOV3 ovarian tumors co-engrafted with human M2 macrophages. While non-significant, due to presence of one outlier mouse failure, F05 again showed additive effects with mAb therapy compared with mAb therapy alone. Importantly, this is the first time a SIRPα specific antibody has shown enhanced efficacy with mAb therapy in the context of true solid tumor model.

While several SIRPα-targeting mAb’s have been described in the pre-clinical setting, F05 has distinct advantages compared to each of them. Two of these agents, KWAR23 and 1H9, do not demonstrate specificity for SIRPα, whereas BR105, AL008 and BYON4228 were shown to specifically bind SIRPα [13–15]. However, BR105 was only shown to have in vivo efficacy in lymphoma models [14]. Additionally, AL008 did not demonstrate significant in vivo efficacy alone in humanized mouse models [13]. BYON4228 demonstrated anti-tumor effects *in vivo*, but only in the context of a Raji lymphoma model in combination with Rituximab [15]. F05 specificity towards SIRPα provides a distinct advantage over 1H9 and KWAR23, the former of which has already been discontinued in the clinic. While Andrejeva *et* al show their antibodies, do not inhibit T cell proliferation, they did not present comparison in vivo efficacy data [21]. Furthermore, BR105 and BYON4228 antibodies were tested in hematopoietic cancer Raji lymphoma, while F05 has therapeutic effects as well in in the context of a solid tumor. BI 765063 and BI770371 have advanced to phase I trials, but BI 765063 importantly only binds SIRPα variant V1, limiting the patent responding pool [22]. Lastly, AL008 was proven effective in a syngeneic setting and not in a human xenograft model. Therefore, F05 warrants further development for clinical application.

## Supporting information

S1 File Additional Figures to support paper. (PPTX)

S2 File Raw data for paper figures, as needed. (XLSX)

## Abbreviations

ELISA: enzyme-linked immunosorbent assay
PBS: phosphate buffered saline
RT: room temperature
PBMC: peripheral blood mononuclear cells
OE: overexpressing
WT: wild type
