# Supplementary material for: A human antibody specific for SIRPα reprograms macrophages and promotes antibody mediated anti-cancer activity
Source: PLoS One. 2025 May 23;20(5):e0321169. doi: 10.1371/journal.pone.0321169 (PMC12101677; doi:10.1371/journal.pone.0321169)
Supplement: S1 File — (PPTX) [file pone.0321169.s001.pptx]

## Slide 1
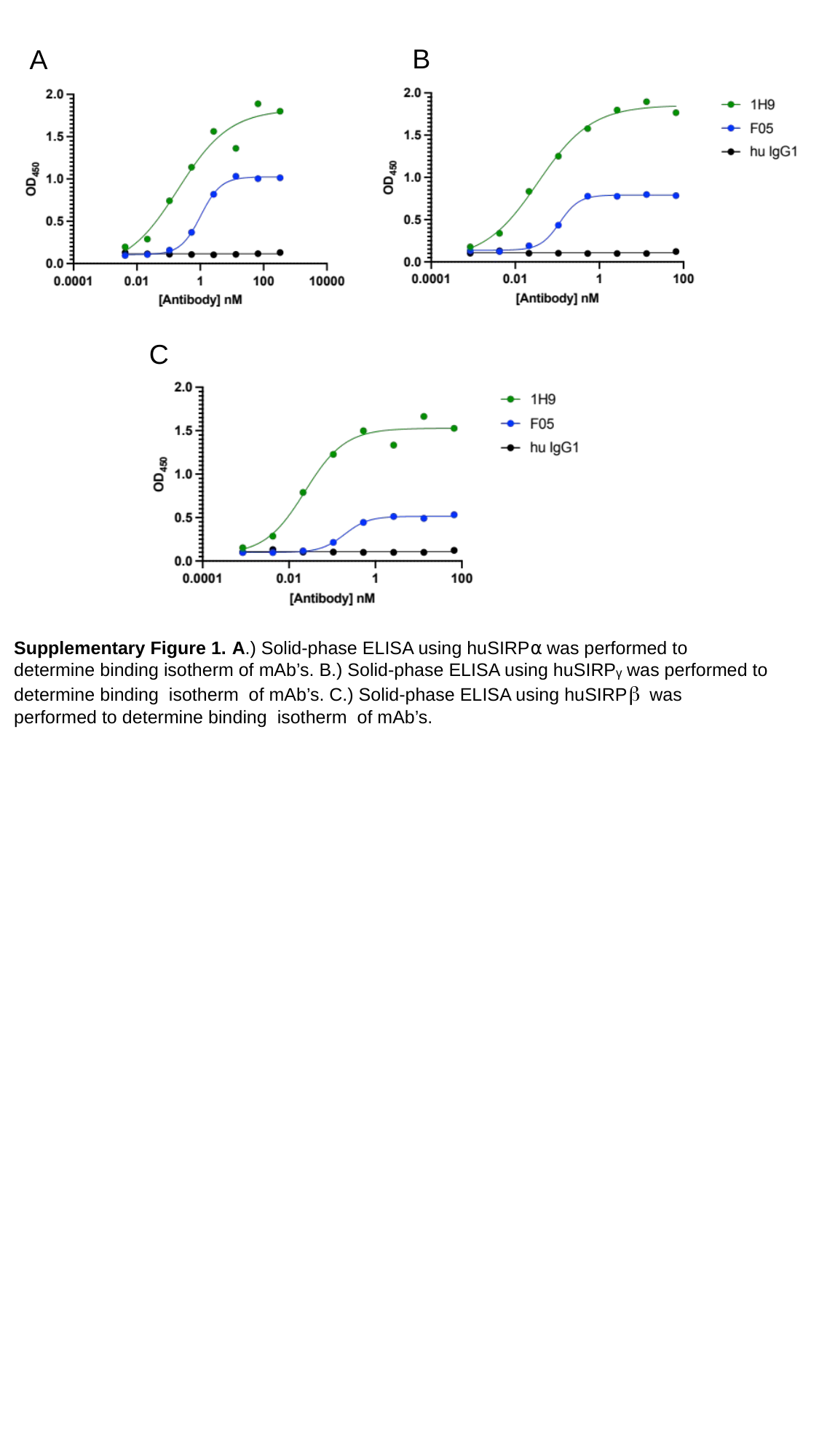

B
A
C
Supplementary Figure 1. A.) Solid-phase ELISA using huSIRP⍺ was performed to determine binding isotherm of mAb’s. B.) Solid-phase ELISA using huSIRPᵧ was performed to determine binding isotherm of mAb’s. C.) Solid-phase ELISA using huSIRPβ was performed to determine binding isotherm of mAb’s.

## Slide 2
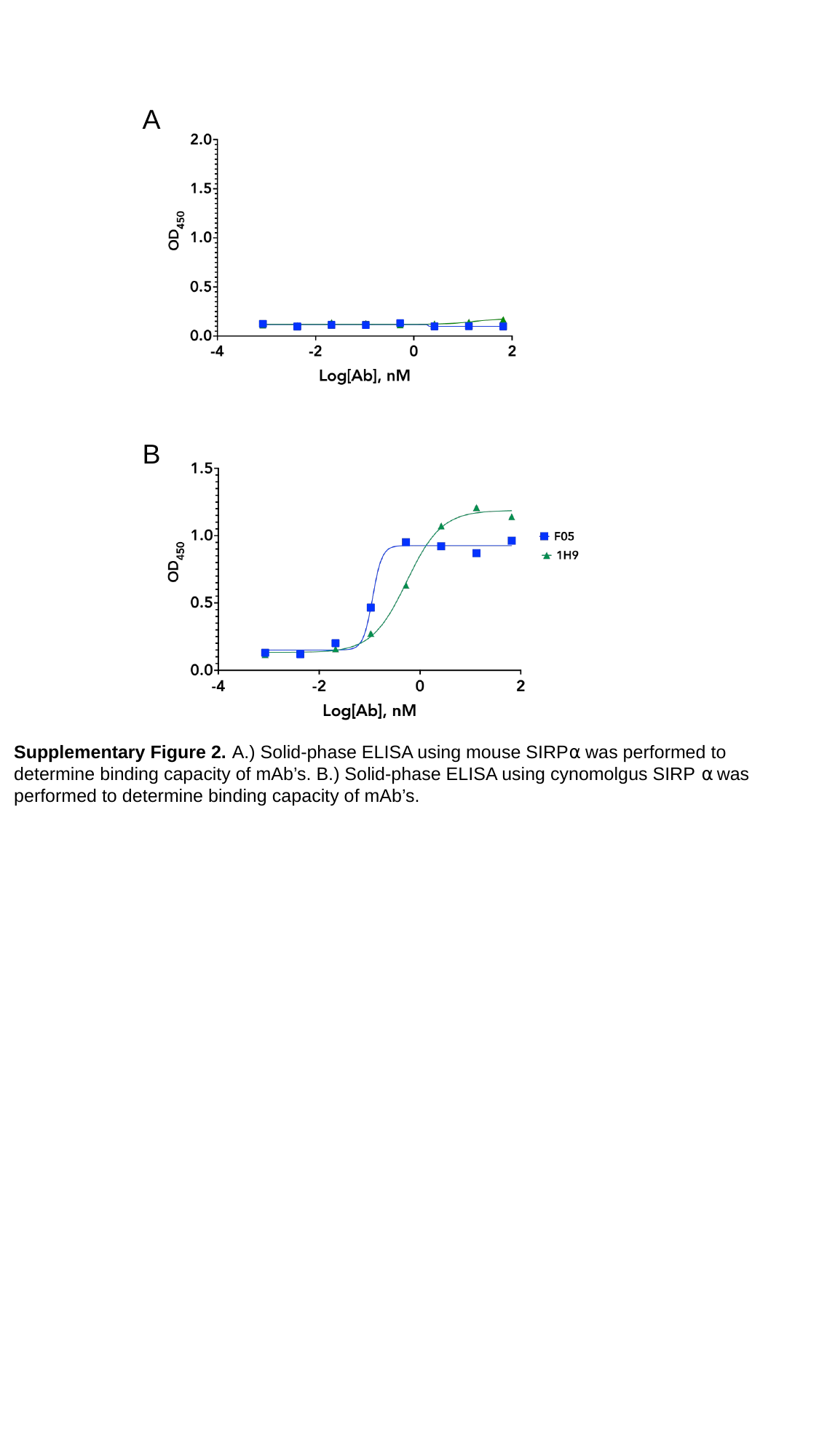

A
B
Supplementary Figure 2. A.) Solid-phase ELISA using mouse SIRP⍺ was performed to determine binding capacity of mAb’s. B.) Solid-phase ELISA using cynomolgus SIRP ⍺ was performed to determine binding capacity of mAb’s.

## Slide 3
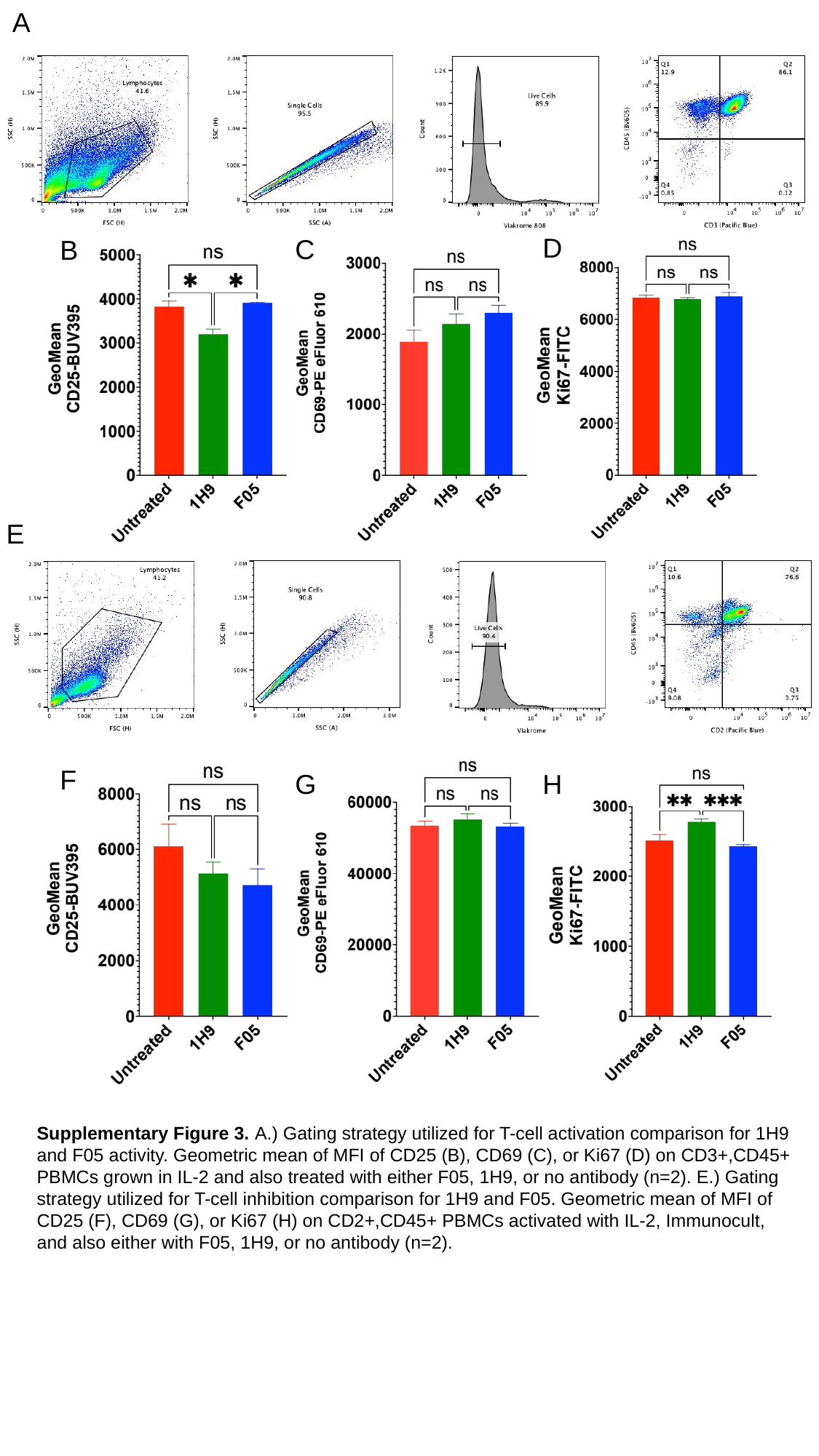

A
D
C
B
E
F
G
H
Supplementary Figure 3. A.) Gating strategy utilized for T-cell activation comparison for 1H9 and F05 activity. Geometric mean of MFI of CD25 (B), CD69 (C), or Ki67 (D) on CD3+,CD45+ PBMCs grown in IL-2 and also treated with either F05, 1H9, or no antibody (n=2). E.) Gating strategy utilized for T-cell inhibition comparison for 1H9 and F05. Geometric mean of MFI of CD25 (F), CD69 (G), or Ki67 (H) on CD2+,CD45+ PBMCs activated with IL-2, Immunocult, and also either with F05, 1H9, or no antibody (n=2).

## Slide 4
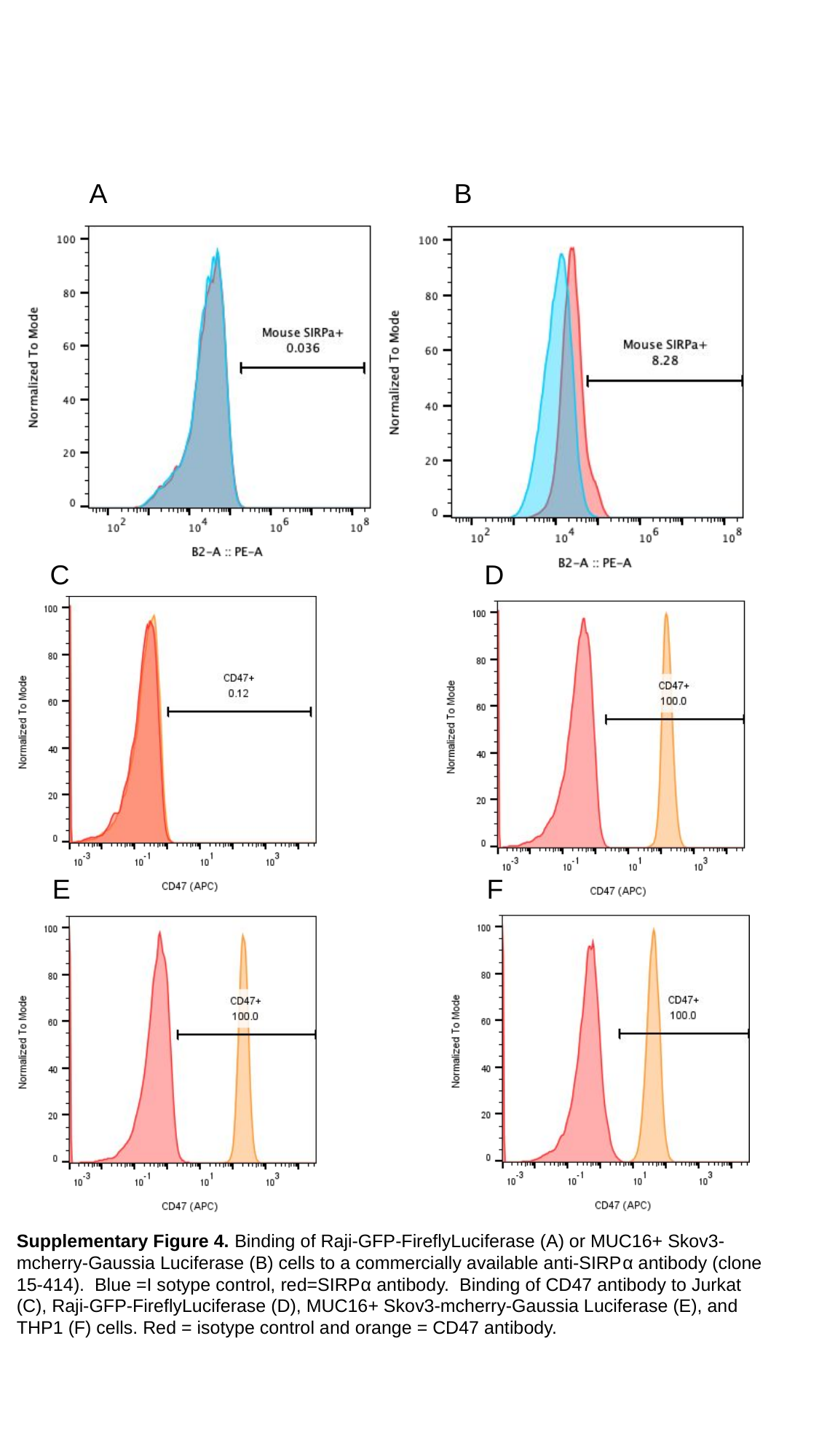

A
B
C
D
E
F
Supplementary Figure 4. Binding of Raji-GFP-FireflyLuciferase (A) or MUC16+ Skov3-mcherry-Gaussia Luciferase (B) cells to a commercially available anti-SIRPα antibody (clone 15-414). Blue =I sotype control, red=SIRPα antibody. Binding of CD47 antibody to Jurkat (C), Raji-GFP-FireflyLuciferase (D), MUC16+ Skov3-mcherry-Gaussia Luciferase (E), and THP1 (F) cells. Red = isotype control and orange = CD47 antibody.

## Slide 5
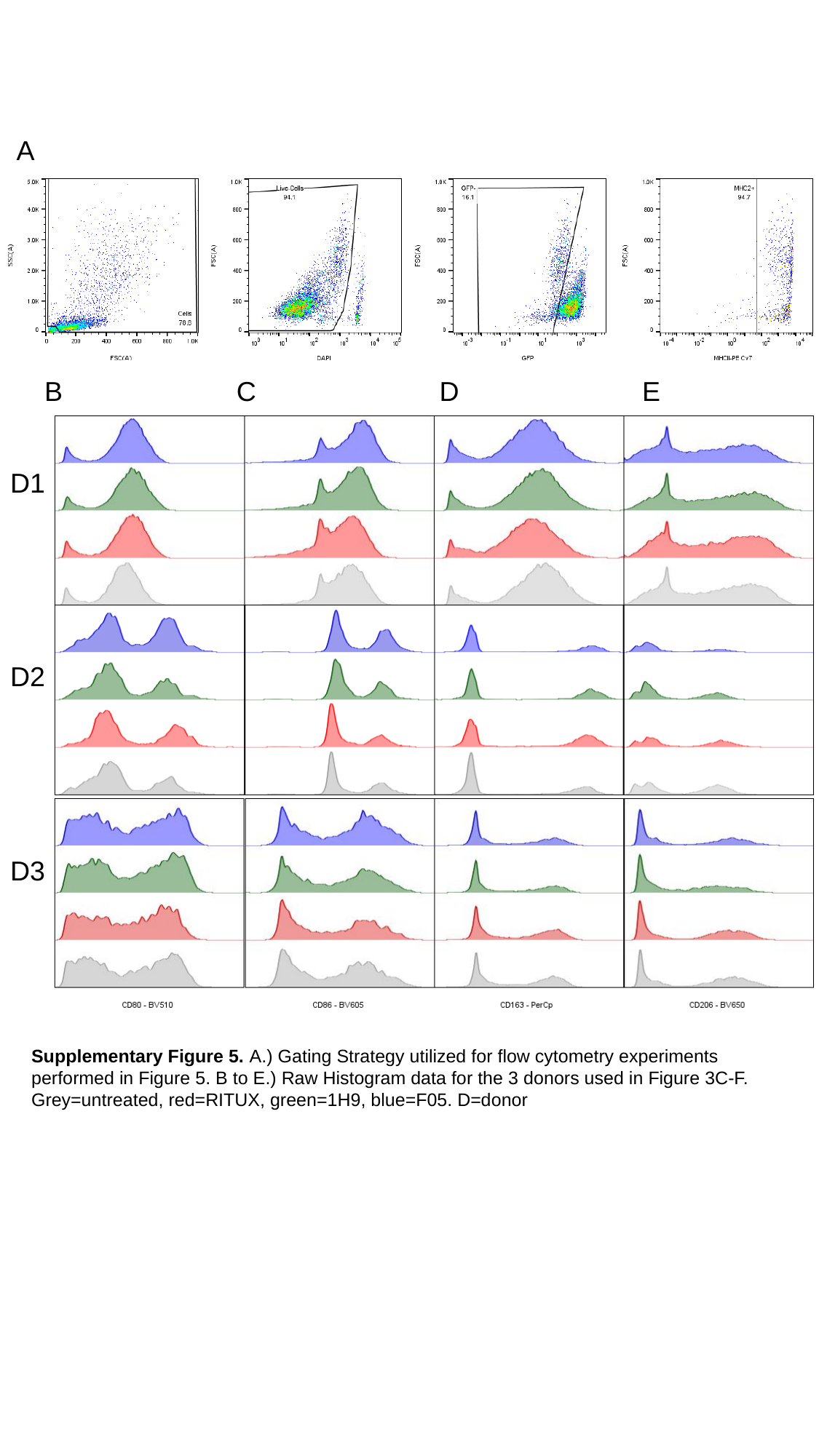

A
B
C
D
E
D1
D2
D3
Supplementary Figure 5. A.) Gating Strategy utilized for flow cytometry experiments performed in Figure 5. B to E.) Raw Histogram data for the 3 donors used in Figure 3C-F. Grey=untreated, red=RITUX, green=1H9, blue=F05. D=donor

## Slide 6
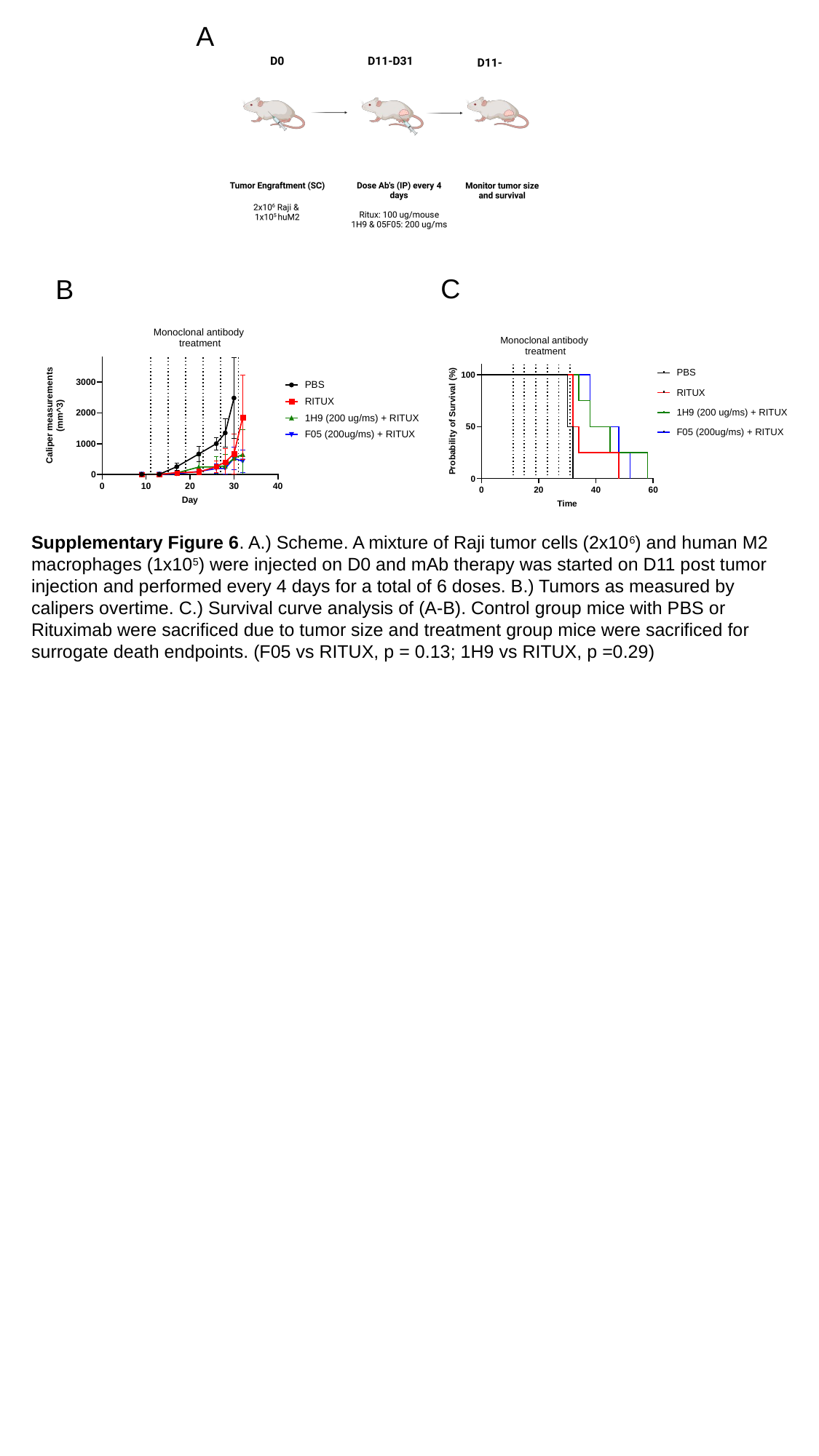

A
C
B
Supplementary Figure 6. A.) Scheme. A mixture of Raji tumor cells (2x10^6^) and human M2 macrophages (1x10^5^) were injected on D0 and mAb therapy was started on D11 post tumor injection and performed every 4 days for a total of 6 doses. B.) Tumors as measured by calipers overtime. C.) Survival curve analysis of (A-B). Control group mice with PBS or Rituximab were sacrificed due to tumor size and treatment group mice were sacrificed for surrogate death endpoints. (F05 vs RITUX, p = 0.13; 1H9 vs RITUX, p =0.29)
